# Interventions to improve the psychosocial outcomes of individuals with lymphedema: a systematic review

**DOI:** 10.1007/s00520-026-10972-9

**Published:** 2026-07-03

**Authors:** Sarah Stolker, Shuya Yin, Wendy Auslander

**Affiliations:** 1 Bursky School of Public Health, Washington University in St. Louis, St. Louis, MO USA; 2 Brown School of Social Work, Washington University in St. Louis, St. Louis, MO USA

**Keywords:** Psychosocial interventions, Mental health, Lymphedema, Supportive care, Systematic review

## Abstract

**Purpose:**

Lymphedema is a common and enduring consequence of cancer treatment with substantial physical and psychosocial morbidity, yet research predominantly focuses on physical outcomes. This systematic review examines psychosocial interventions for individuals with lymphedema, describing the intervention characteristics, methodological quality, and strength of the evidence.

**Methods:**

A systematic literature search was conducted of studies published through November 2025. Empirical interventions with psychosocial components and outcomes for individuals with lymphedema were eligible. Methodological quality was assessed. Strength of the evidence was determined by combining quality with intervention effectiveness.

**Results:**

Ten studies met inclusion criteria, including mind–body (*n* = 3), education and support (*n* = 3), and physical activity (*n* = 4) intervention models, and most delivered following completion of decongestive therapy. Psychosocial outcomes included mental health (*n* = 8) and quality of life (*n* = 6). Methodological rigor was mixed, with MQRS scores ranging from 5–13 (of 14). Interventions delivered in combined in-clinic and remote settings demonstrated stronger evidence for improving mental health outcomes than either setting alone.

**Conclusion:**

Few empirical evaluations exist targeting the psychosocial needs of individuals with lymphedema, particularly during intensive phases of decongestive therapy. Heterogeneity in intervention models, outcome measures, and treatment timing limits cross-study comparisons; however, hybrid delivery approaches show promise for improving psychosocial outcomes. Greater integration of psychosocial care across the lymphatic care continuum, supported by standardized outcome measures, may strengthen supportive care for individuals living with lymphedema.

**Supplementary Information:**

The online version contains supplementary material available at 10.1007/s00520-026-10972-9.

## Introduction

Lymphedema is a chronic and potentially debilitating condition caused by damage or insufficiency of the lymphatic system, leading to the accumulation of protein-rich fluid in the tissues. It affects over 250 million people worldwide and has been identified as a major public health issue [1, 2]. Lymphedema is classified as primary, arising from intrinsic abnormalities in the development of lymphatic tissues [3], or secondary, occurring when lymph vessels are damaged or removed. Secondary lymphedema can result from a wide range of etiologies, including cancer treatment, trauma, and infection [2, 4]. In developed countries, cancer-related lymphedema (CRL) is among the most prevalent forms, occurring as a consequence of lymph node removal and radiation therapy across multiple cancer types [2]. Lymphedema arising from infectious etiologies such as lymphatic filariasis and podoconiosis represents a distinct clinical and psychosocial experience due to cultural and environmental factors [5]. Given these differences, this systematic review examines psychosocial interventions and outcomes in non-infectious lymphedema, with particular relevance to cancer populations.

The physical symptoms of lymphedema, such as swelling, pain, tenderness, soreness, aching, heaviness, tightness, and numbness, are progressive, leading to worsening functional mobility and increased infection risk [6–11]. Beyond physical symptoms, the psychosocial impacts of breast cancer-related lymphedema (BCRL) have been well-documented and substantial [2, 6, 7, 9, 11]. Reduced psychosocial well-being in BCRL has been linked to symptoms of anxiety/stress, depression, emotional distress, fatigue, body image disturbances, impaired mobility, and decreased participation in social activities [9, 12, 13]. Similarly, women with lower extremity lymphedema secondary to treatment for gynecological cancer have experienced psychological sequelae, including decreased self-confidence, loss of identity, and social isolation [14, 15]. The complex interplay between physical and psychosocial symptoms is evidenced by negative behavioral consequences such as decreased participation in recreational activities, difficulties in social life, work-related challenges, and problems in close relationships [7, 9, 16]. Moreover, cross-cultural evidence has confirmed these findings among individuals with lymphedema, including feelings of frustration and isolation, body image burden, and barriers to occupational reintegration [5, 6, 17]. Additionally, individuals with cancer who develop lymphedema also have reported lower quality of life, poorer sleep quality, and greater fear of cancer recurrence than their counterparts without lymphedema [15]. Experiences of individuals with primary or non-cancer-related secondary lymphedema have been less well-documented but are compounded by profound diagnostic and treatment delays, resulting in even greater psychosocial morbidity [16, 18, 19].

To date, four reviews have examined psychosocial impacts of lymphedema, including one focused on cancer-related lymphedema [11], one examining lymphedema across mixed etiologies [6], one addressing primary lymphedema [19], and one addressing lymphatic filariasis [5]. A 2013 systematic review by Fu and colleagues [6] that summarized the evidence and identified factors influencing psychosocial impacts found that lymphedema characteristics such as stage, location, and pain were associated with greater anxiety and psychological distress in BCRL. Burden of time-intensive self-care, the unexpected and incurable nature of the condition, and associated social and financial strain also exacerbated emotional distress. Eaton and colleagues [11] updated these findings and added economic burdens, lack of social support, impaired sexuality, and unmet educational needs to the psychosocial impacts of BCRL. Beyond cancer-related lymphedema, a systematic review of negative psychosocial effects of primary lymphedema described feelings of stress, embarrassment, body image disturbances, low self-esteem, relationship difficulties, and symptoms of depression [19]. In their systematic review, Vasconez-Gonzalez and colleagues found that lymphatic filariasis poses a profound psychosocial burden, frequently exacerbated by intense societal rejection, marginalization and stigma [5]. The 2023 Consensus Document of the International Society of Lymphology called lymphedema a chronic, generally incurable condition requiring lifelong care and attention, along with psychosocial support [4]. Despite strong recommendations for psychosocial support, findings from the 2024 Lymphatic Education and Research Network’s (LE&RN) registry report emphasized critical gaps in the lymphatic care continuum and the need for interventions to address the substantial unmet psychosocial needs in this population [18].

Despite the profound psychosocial impacts associated with lymphedema, no systematic reviews to date have focused specifically on evaluating interventions aimed at *improving* the psychosocial outcomes of individuals with lymphedema. Likewise, no studies have examined the methodological rigor of these evaluation studies. The current study addressed this gap by conducting a systematic review of the evidence of psychosocial interventions for individuals with lymphedema. To deepen our knowledge of interventions targeting the psychosocial aspects of lymphedema, this systematic review addressed the following research questions: 1) What types of psychosocial interventions have been used to treat lymphedema (i.e., intervention models, duration, and setting) and what stage of lymphedema treatment were the interventions designed for? 2) What were the most common psychosocial outcomes measured? 3) What was the methodological quality of these studies? and 4) What types of interventions were most effective when considering the study’s methodological rigor, that is, what is the strength of the evidence supporting these interventions?

## Methods

### Design

A systematic review (Open Science Framework ID: osf.io/2szyv) was conducted in accordance with the Preferred Reporting Items for Systematic Reviews and Meta-Analyses (PRISMA) 2020 [20].

### Search strategy

Prior to conducting the literature search, the author developed search protocols, inclusion criteria, and designed a data extraction table to document details, including authors, publication year, study purpose, intervention duration, intervention location, study design, theoretical foundation, and outcomes measured. Search terms and databases were developed after consulting with a university-based librarian. Table 1 displays the search terms and databases used for the current systematic review. The literature search was initially conducted on January 24, 2025, and subsequently updated on December 3, 2025, with the latter representing the final search date.

**Table 1:** Systematic review search terms and databases

**Databases Searched:** MEDLINE, Academic Search Complete, America: History & Life, APA PsycInfo, CINAHL Plus, Communication Source, Family & Society Studies Worldwide, Gender Studies Database, Global Health,orMilitary & Government Collection, SocINDEX with Full Text
**Search Terms:** TI (intervention* OR postintervention OR postvention OR program* OR initiative* OR project* OR pilot OR service* OR support* OR "self help group*" OR psychotherap* OR counsel* OR "family therapy" OR "couples therapy" OR mindfulness OR yoga OR pilates OR "breathing exercise*" OR "tai chi" OR "educational group" OR curricul* OR "patient handout*" OR "educational material*" OR "instructional material*" OR psychoeducation*) OR AB (intervention* OR postintervention OR postvention OR program* OR initiative* OR project* OR pilot OR service* OR support* OR "self help group*" OR psychotherap* OR counsel* OR "family therapy" OR "couples therapy" OR mindfulness OR yoga OR pilates OR "breathing exercise*" OR "tai chi" OR "educational group" OR curricul* OR "patient handout*" OR "educational material*" OR "instructional material*" OR psychoeducation*) OR SU (intervention* OR postintervention OR postvention OR program* OR initiative* OR project* OR pilot OR service* OR support* OR "self help group*" OR psychotherap* OR counsel* OR "family therapy" OR "couples therapy" OR mindfulness OR yoga OR pilates OR "breathing exercise*" OR "tai chi" OR "educational group" OR curricul* OR "patient handout*" OR "educational material*" OR "instructional material*" OR psychoeducation*)
AND TI ("quality of life" OR "QOL" OR psychosocial* OR "psycho social*" OR anxiety OR depression OR "body image" OR "social interactions" OR anger OR "self identity" OR hopelessness OR loneliness OR "mental health" OR "mental well being" OR "mental wellbeing" OR distress OR frustrat*) OR AB ("quality of life" OR "QOL" OR psychosocial* OR "psycho social*" OR anxiety OR depression OR "body image" OR "social interactions" OR anger OR "self identity" OR hopelessness OR loneliness OR "mental health" OR "mental well being" OR "mental wellbeing" OR distress OR frustrat*) OR SU ("quality of life" OR "QOL" OR psychosocial* OR "psycho social*" OR anxiety OR depression OR "body image" OR "social interactions" OR anger OR "self identity" OR hopelessness OR loneliness OR "mental health" OR "mental well being" OR "mental wellbeing" OR distress OR frustrat*)
AND (TI (lymphedema OR AB lymphedema OR SU lymphedema)
AND applied limits of Peer-reviewed, English language

### Selection criteria

Studies were eligible for inclusion if they met the following criteria: (1) participants diagnosed with non-infectious lymphedema, (2) published in English prior to Dec 1, 2025, (3) empirically based evaluations of interventions with psychosocial components and psychosocial outcomes, (4) quantitative statistical analyses, and (5) experimental or quasi-experimental research designs. Psychosocial interventions were defined as activities targeting behavioral, cognitive, emotional, or social factors to improve functioning and well-being [21]. Psychosocial outcomes were defined as measures that assessed the emotional, cognitive, behavioral, interpersonal, and social aspects of health and well-being [22]. Studies were excluded from the review if quality of life (QoL) was the only outcome measure used to evaluate the interventions, since QoL measures often do not adequately capture psychosocial domains. Second, studies with participants diagnosed with lymphedema resulting from filariasis or podoconiosis were also excluded due to their unique etiology, treatment, and psychosocial experiences.

### Appraisal of methodological quality

The methodological quality of the studies was assessed using an adapted version of the Methodological Quality Rating Scale (MQRS) by Miller et al. [23], displayed in Table 2. The MQRS has been successfully used in prior systematic reviews [24, 25]. To adapt the MQRS for this study, the present study made the following modifications: (1) *Statistical power* was added to determine whether the study had adequate sample size and statistical power. (2) *Theoretical foundation* was operationalized to include any reference to the theoretical model or framework. (3) The *Dosage* criterion was modified so that studies with fewer than 10 sessions (< 10) were scored as 0, and those delivering 10 or more sessions (≥ 10 based on prior studies in psychotherapy identifying ~ 8–11 sessions as the median range to observe reliable or clinically significant change) [26]. The possible total scale scores for the adapted MQRS ranged from 0 (low) to 14 (high), with higher scores indicating higher methodological quality. The mean total scale scores for the sample were used to determine which studies had higher or lower methodological quality. Each study’s rigor was independently rated by two authors (S.S. and S.Y.). Disputes were resolved by discussion or, if necessary, by consulting a third author (W.A.).

**Table 2:** Adapted MQRS rating scale

Methodological Criteria	Rating (points)^a^
1. Study Design	0 = pre/post 1 = quasi-experimental 2 = randomized controlled trial
2. Theoretical Foundation	0 = Treatment not theoretically based or not reported 1 = Theoretical basis discussed or referenced
3. Treatment Integrity	0 = No standardization of intervention cited or assessment of fidelity 1 = Standardized
4. Measures	0 = reliability and validity not reported or inadequate 1 = Reliability and validity of measures clearly adequate
5. Length of follow up	0 = No follow-up 1 = Less than the intervention phase 2 = Equal to or greater than the intervention phase
6. Dosage	0 = < 10 sessions 1 = > or equal to 10 sessions
7. Dropouts/attrition	0 = Dropouts neither discussed nor accounted for 1 = Intervention dropouts enumerated and discussed
8. Blind follow-up	0 = Post-test or follow-up conducted by non-blind person or did not specify 1 = Follow-up by person blind to participants’ treatment condition
9. Analysis	0 = Inappropriate statistical analysis conducted or did not use all available data 1 = Appropriate statistical analyses
10. Statistical Power	0 = Clearly inadequate power due to small sample size or dropout 1 = Adequate power with adequate sample size
11. Multi-Site	0 = Single site study 1 = Parallel replications at two or more sites
12. Generalizability	0 = No discussion of generalizability of findings 1 = Discussion of generalizability of results (e.g., sample characteristics, site, treatment)

Source: [23]

^a^ Possible summary or total scores range from 0 (low) to 14 (high)

### Study effectiveness and rating the strength of the evidence

The effectiveness of each intervention was evaluated by outcome according to whether the results of the analyses were significant or not significant, including the direction of the effects for the primary psychosocial outcomes. Additionally, the strength of the evidence was rated by taking into account the methodological rigor of the study. Studies that demonstrated “strong evidence” were those studies that resulted in a significant outcome in the hypothesized direction and high methodological rigor (i.e., MQRS total scores greater than or equal to the mean score of the sample). Interventions were considered “promising evidence” if the study had a significant outcome but low rigor (i.e., MQRS total scores below the mean). Studies were considered to have “weak evidence” if they had non-significant outcomes and either low or high methodological rigor.

## Results

### Search and selection of studies

**Fig. 1:**
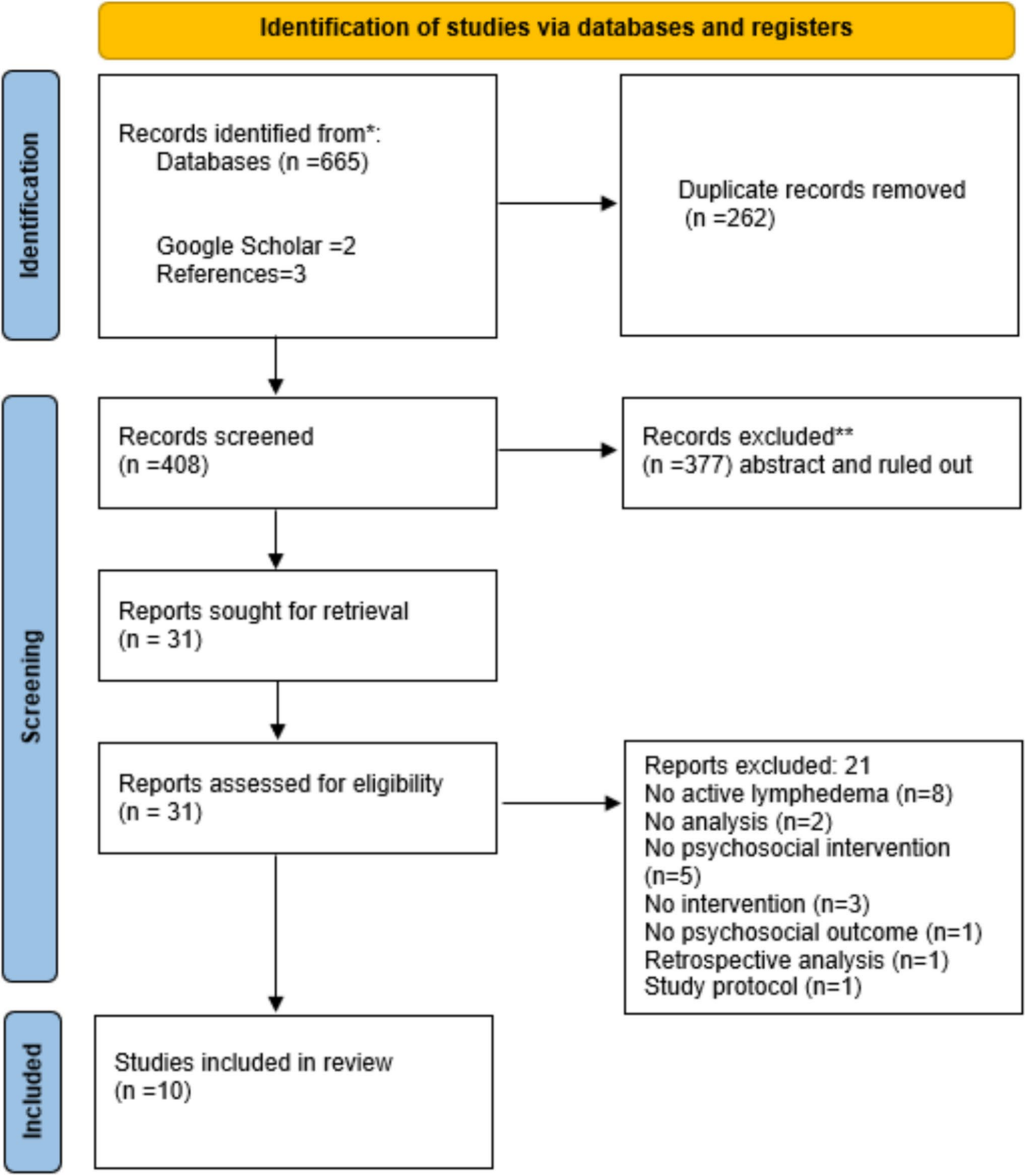
PRISMA 2020 Flow Diagram of the search strategy and study selection process. PRISMA: Preferred Reporting Items for Systematic Reviews and Meta-Analyses

As shown in Fig. 1, the systematic search resulted in 665 records, and the subsequent manual search identified 5 potentially eligible studies. Following duplicate removal, the titles and abstracts of 408 were screened, with subsequent assessment of 31 full texts. Of those 31 studies, 21 were excluded for not meeting study criteria, resulting in a final sample of 10 intervention studies for the present systematic review.

### Study and participant characteristics

Characteristics of the ten studies are shown in Table 3 and include authors, study design, country of intervention location, target population, intervention model, sample size/power, psychosocial outcomes, QOL and mental health measures, and whether the study was a pilot study. Nine studies were randomized controlled trials, and one was quasi-experimental. Studies were conducted in five countries: Australia (*n* = 2), Austria (*n* = 1), Iran (*n* = 3), Japan (*n* = 2), and the USA (*n* = 2). Populations were predominantly breast cancer-related lymphedema (BCRL; *n* = 8), with one study on gynecological lower extremity lymphedema (*n* = 1) and one study on mixed lymphedema or lipedema in adults undergoing inpatient rehabilitation (*n* = 1). Total sample sizes for the studies ranged from 28 to 304 participants, with six studies including fewer than 50 participants.

**Table 3:** Intervention characteristics summary (*N* = 10)

Authors	MQRS	Design	Country	Population^a^	Model	Timing	Sample (I vs C)	Psychosocial Outcomes^b^	Measures	Pilot	Ref
Abbasi et al. 2018	11	Quasi-Exp	Iran	BCRL	Mind/Body	Phase 1	16 vs 15	Anxiety, Depression	HADS	No	[27]
Arinaga et al. 2019	8	RCT	Japan	BCRL	Physical Activity	Phase 2	22 vs 21	BCRL symptoms, QOL, self-care	SF-8, Patient-rated scale	Yes	[28]
Loibnegger-Traubnig et al. 2023	9	RCT	Austria	Lymphedema	Mind/Body	Phase 1	36 vs 35	Stress, HRV	PSS-10, TICS	No	[29]
Loudon et al. 2014	12	RCT	Australia	BCRL	Physical Activity	Phase 2	15 vs 13	Fatigue, QOL	LYMQOL, Patient-rated scale	Yes	[30]
McClure et al. 2010	11	RCT	USA	BCRL	Physical Activity	Phase 2	16 vs 16	Mood, QOL	SF-36, BDI-II	No	[31]
Okutsu and Koiyabashi 2014	5	RCT	Japan	Leg Lymphedema	Education & Support	Phase 2	15 vs 15	Mental health, QOL, Self-care	FACT-G, MHP	No	[32]
Omidi et al. 2020	12	RCT	Iran	BCRL	Education & Support	Phase 1	35 vs 35 vs 35	Fear of Ca recurrence, QOL	LLIS, FoPQ-SF	No	[33]
Pasyar et al. 2019	8	RCT	Iran	BCRL	Physical Activity	Phase 2	20 vs 20	Fatigue, Insomnia, QOL	EORTC QLQ-C30, Patient-rated scale	Yes	[34]
Ridner et al. 2020	12	RCT	USA	BCRL	Education & Support	Phase 2	80 vs 80	Bio-behavioral symptoms, Well-being, Coping/Stress, Mood	POMS-SF, PSS-4, MOS, Brief COPE, LSIDS-A	No	[35]
Sherman et al. 2018	13	RCT	Australia	BC/BCRL	Mind/Body	No restrictions	149 vs 155	Body-image, Self-compassion, Anxiety, Depression	SCS-SF, DASS, BIS	No	[36]

^a^Abbreviations: *BC* breast cancer, *BCRL* Breast cancer-related lymphedema, *NA* not applicable, *QOL* quality of life, *RCT* randomized controlled trial

^b^Abbreviation of Measures *BDI-II* Beck Depression Inventory-II, *BIS* Body Image Scale, *Brief* *COPE* Coping Orientation to Problems Experienced Inventory, *DASS* Depression, Anxiety and Stress Scales, *EORTC QLQ-C30* European Organisation for Research and Treatment of Cancer Quality of Life Questionnaire Core 30, *FACT-G* Functional Assessment of Cancer Therapy-General, *FoPQ-SF* Fear of Progression Questionnaire- Short Form, *HADS* Hospital Anxiety and Depression Scale, *LLIS* Lymphedema Life Impact Scale, *LSIDS-A* Lymphedema Symptom Intensity and Distress Scale-Arm *LYMQOL* Lymphedema Quality of Life Questionnaire, *MHP* Mental Health Pattern, *MOS* Medical Outcomes Social Support Survey, *POMS-SF* Profile of Mood States- Short Form, *PSS-4* Perceived Stress Scale-4 item, *SCS-SF* Self Compassion Scale Short Form, *SF-8* Short Form 8 Health Survey, *MQRS* Methodological Quality Rating Scale, *SF-36* Short Form 36 Health Survey, *TICS* Trier Inventory for Chronic Stress

### Intervention models, stage and length of treatment

To characterize the types of psychosocial interventions used to treat lymphedema, interventions were grouped into three mutually exclusive categories. Each study was assigned to a single category based on the primary intervention components and their theoretical underpinning: Mind/Body (*n* = 3), Education & Support (*n* = 3), and Physical Activity (*n* = 4). Mind/Body (MB) interventions [27, 29, 36] incorporated psychological approaches such as relaxation, self-compassion, and cognitive-based stress management. Education and support (ES) interventions [32, 33, 35] integrated patient-centered education and support to improve adherence and symptom management. Physical activity (PA) interventions [28, 30, 31, 34], were primarily movement/exercise interventions that assessed both physical and psychosocial outcomes.

### Outcomes and measures

Most of the studies assessed both physical and psychosocial outcomes. The most common psychosocial outcomes were quality of life (*n* = 6) and mental health-related outcomes (*n* = 8), which included depression, anxiety/stress, fear of cancer recurrence, coping, self-compassion, body image distress, and insomnia/fatigue. Non-mental health psychosocial outcomes, such as symptom burden and perceived self-management, were assessed in too few studies to allow for meaningful comparison. Quality of life (QoL) measures were heterogeneous: two studies used general QoL instruments [28, 31], two used cancer-specific measures [32, 34], and two used lymphedema-specific scales [30, 33].

### Intervention delivery sites and timing

The interventions in this review varied in terms of their site of delivery. Some were conducted at rehabilitation centers [27, 29, 33] (*n* = 3), specialized medical clinics [28, 30, 31, 34] (*n* = 4) or remotely via phone or web [32, 35, 36] (*n* = 3), with six studies combining in-clinic and remote components (IC&R) [27, 30–34]. Interventions also varied in the timing or stage of lymphedema treatment: Mind–Body and Education and Support interventions were most often delivered during the intensive phase (Phase 1) [27, 29, 33] (*n* = 3), while Physical Activity interventions typically targeted ongoing Phase 2 self-management [28, 31] (*n* = 2). Four interventions were designed as post-treatment supplements [30, 32, 34, 35], and one intervention had no restrictions on timing or concomitant care [36] (*n* = 1). Intervention duration ranged from a single session (therapeutic writing; [36] to intensive clinical programs lasting 3–8 weeks. These included the following modalities: relaxation exercises [27], mindfulness training [29], educational sessions [33], yoga sessions [30, 34], and supervised exercise sessions over 5 weeks [31]. Two studies delivered longer-term programs, lasting 3 to 6 months, such as daily holistic exercise [28] and continuous self-management with technology-assisted support [32].

### Methodological quality ratings

Results of the MQRS ratings for each study, by item and total scale scores, are displayed in Table S1. Total scale scores for each study ranged from 5 to 13, with a mean score of 10.1 (SD = 2.51). Using a mean split, six of 10 studies were categorized as higher methodological rigor (MQRS ≥ 10.1), and four as lower rigor (MQRS < 10.1) (Figure S2). Several methodological strengths were evident across studies: 90% of studies (*n* = 9) were randomized controlled trials, demonstrating rigor of study design in this sample. All ten studies were theoretically informed and used valid and reliable measures. Half of the studies (50%) included follow-up assessments equal to or longer than the intervention period. The most common methodological areas for improvement across the studies were small sample sizes; the majority of studies (*n* = 8) included ≤ 36 participants in each of control/treatment conditions, increasing the likelihood of inadequate statistical power. Additionally, 80% (*n* = 8) of the studies were conducted at a single site rather than multiple sites, and 50% (*n* = 5) did not discuss the generalizability of the findings.

### Comparisons of study effectiveness by intervention model

In order to examine what types of interventions were most effective, taking into account the studies’ methodological rigor, analyses were conducted for the two major categories of outcomes (i.e., Quality of Life, and Mental Health) in the current review. As shown in Table 4, six studies included quality of life as an outcome variable. Of those six studies, five studies reported significant improvements in QoL. Yet, when taking into account the methodological rigor of the studies, two studies demonstrated the strongest evidence for improving the quality of life among participants; one tested an Education & Support intervention [33], and one tested a Physical Activity intervention [31].

**Table 4:** Comparisons of study effectiveness by intervention model

Intervention Model Comparison					
Authors (Year)	Intervention Model	MQRS Rating	Methodological Rigor	Outcome Significance	Strength of Evidence
Quality of Life Outcomes					
Omidi et al. (2020) [33]	Education & Support	12	High	Significant improvements	Strong
Okutsu and Koiyabashi (2014) [32]	Education & Support	5	Low	Significant improvements	Promising
McClure et al. (2010) [31]	Physical Activity	11	High	Significant improvements	Strong
Arinaga et al. (2019) [28]	Physical Activity	8	Low	Significant improvements	Promising
Pasyar et al. (2019) [34]	Physical Activity	8	Low	Significant improvements	Promising
Loudon et al. (2014) [30]	Physical Activity	12	High	Not significant	Weak
Mental Health Outcomes					
Authors (Year)	Intervention Model	MQRS Rating	Methodological Rigor	Outcome Significance	Strength of Evidence
Omidi et al. (2020) [33]	Education & Support	12	High	Significant improvements	Strong
Ridner et al. (2020) [35]	Education & Support	12	High	Significant improvements	Strong
Okutsu and Koiyabashi (2014) [32]	Education & Support	5	Low	Significant improvements	Promising
McClure et al. (2010) [31]	Physical Activity	11	High	Significant improvements	Strong
Arinaga et al. (2019) [28]	Physical Activity	8	Low	Significant improvements	Promising
Abbasi et al. (2018) [27]	Mind/Body	11	High	Significant improvements	Strong
Sherman et al. (2018) [36]	Mind/Body	13	High	Significant improvements	Strong
Loibnegger-Traußnig et al. (2023) [29]	Mind/Body	9	Low	Not Significant	Weak

As shown in Table 4, the current review included eight studies that examined mental health outcomes. Among those eight studies, the intervention models included: Education & Support (*n* = 3), Physical Activity (*n* = 2), and Mind/Body (*n* = 3). Of those eight studies, seven showed significant improvements in mental health outcomes. Among Education and Support interventions, two of three (67%) provided strong evidence [33, 35]. Likewise, among Mind/Body interventions, two of three (67%) also demonstrated strong evidence [27, 36] for improving mental health outcomes. Finally, among the two Physical Activity interventions with mental health outcomes, one of two (50%) demonstrated strong evidence [31].

### Comparisons of study effectiveness by intervention delivery setting

As shown in Table 5, there were six studies that included QoL outcomes. Of those six, four were in-clinic and remote (IC&R) delivery interventions [30, 31, 33, 34] and two were remote-only delivery interventions [28, 32] (*n* = 2). The analysis indicated that five of the six studies showed significant improvements in QoL outcomes. Yet when taking into account the methodological rigor, only two studies demonstrated strong evidence for improving QoL, and both were IC&R delivery interventions. Although neither of the two remote-only interventions provided strong evidence, both studies [28, 32] demonstrated promising evidence and warrant further testing with more rigorous methods. Among the eight studies that included mental health outcomes, three were implemented in in-clinic and remote settings, one was implemented solely in the clinic, and four were remote interventions. Results indicated that seven of the eight interventions showed significant improvements in mental health outcomes. When considering the methodological rigor of the seven studies, all hybrid delivery interventions demonstrated strong evidence (Table 5). Of the remote-only interventions, two studies provided strong evidence [35, 36], and two provided promising evidence [28, 32]. Notably, all remote-only interventions resulted in significant outcomes, despite half having lower methodological rigor*.* The intervention delivered in-clinic-only (*n* = 1) had non-significant results and thus provided weak evidence for improving mental health outcomes [29]. Overall, these results suggest that interventions that are delivered both in-clinic and remote settings may be most effective for improving mental health outcomes among individuals with lymphedema.

**Table 5:** Comparisons of study effectiveness by delivery setting

Authors (Year)	Intervention Model^a^	MQRS Rating	Methodological Rigor	Outcome Significance	Evidence of Effectiveness
Quality of Life Outcomes					
Omidi et al. (2020) [33]	IC&R	12	High	Significant improvements	Strong
McClure et al. (2010) [31]	IC&R	11	High	Significant improvements	Strong
Pasyar et al. (2019) [34]	IC&R	8	Low	Significant improvements	Promising
Loudon et al. (2014) [30]	IC&R	12	High	Not Significant	Weak
Okutsu and Koiyabashi (2014) [32]	Remote	5	Low	Significant improvements	Promising
Arinaga et al. (2019) [28]	Remote	8	Low	Significant improvements	Promising
Mental Health Outcomes					
Abbasi et al. (2018) [27]	IC&R	11	High	Significant improvements	Strong
Omidi et al. (2020) [33]	IC&R	12	High	Significant improvements	Strong
McClure et al. (2010) [31]	IC&R	11	High	Significant improvements	Strong
Loibnegger-Traußnig et al. (2023) [29]	IC	9	Low	Not Significant	Weak
Ridner et al. (2020) [35]	Remote	12	High	Significant improvements	Strong
Sherman et al. (2018) [36]	Remote	13	High	Significant improvements	Strong
Okutsu and Koiyabashi (2014) [32]	Remote	5	Low	Significant improvements	Promising
Arinaga et al. (2019) [28]	Remote	8	Low	Significant improvements	Promising

^a^*IC* in-clinic only, *IC&R* in-clinic and remote

## Discussion

The current systematic review examined psychosocial interventions for individuals with lymphedema, investigating the types and timing of interventions, the outcomes measured, and their effectiveness, while accounting for methodological rigor.

The findings of this review highlight variability in psychosocial interventions, both in intervention models and in timing. Given that lymphedema is mostly commonly treated in rehabilitation settings, the inclusion of physical activity interventions is not surprising. Despite mounting evidence of lymphedema-related distress prompting recommendations for the development of interventions that target psychosocial functioning, current treatment approaches remain focused on physical endpoints [37, 38]. Historic activity limitations for individuals at risk of lymphedema [11] may also account for continued emphasis on safety, as illustrated by the two yoga-based [30, 34] studies in our sample [39]. Interventions were overwhelmingly conducted within breast cancer lymphedema (BCRL) populations, potentially limiting generalizability to lymphedema associated with other cancers and to non-cancer-related lymphedema, populations that remain underrepresented in the literature [17, 19] despite experiencing additional diagnostic delays and poorer access to treatment [18].

In addition to differences in intervention type, the timing of psychosocial interventions also varied, which may influence both access and effectiveness. Mind–Body and Education and Support interventions were most often delivered in Phase 1, typically concurrent with Complete Decongestive Therapy (CDT), and focused on addressing psychosocial needs through resource building. Ridner and colleagues [35] based their intervention on Lazarus and Folkman’s Stress and Coping model [40], hypothesizing that adaptive changes in appraisals and coping strategies fostered improved self-management adherence and enhanced outcomes. Across the two-phase CDT treatment, several interventions were implemented as post-treatment supplements (*n* = 4). Given that access to lymphedema care remains challenging due to a limited number of lymphedema therapists and treatment centers [18], relying on even a subset of post-treatment interventions could limit timely access to psychosocial support. Additionally, insights from rehabilitation research support early intervention to minimize the development and severity of side effects of chronic disease [41]. Findings from a systematic review of complementary therapies suggest that while interventions during rehabilitation treatment highlight the significance of the therapeutic exchange and practitioner expertise, more evidence is needed regarding timing and outcome efficacy [42]. Taken together, these findings highlight the need for further research on the optimal timing of psychosocial interventions in lymphedema care [43].

Variability in psychosocial outcomes limits the ability to compare results, with QoL and mental health being the most commonly measured constructs. This heterogeneity likely reflects differences in the theoretical focus of interventions and the hypothesized mechanisms of action. For example, Omidi et al. utilized an Education and Support model to improve patient motivation and adherence in lymphedema self-management, measuring QoL with the Lymphedema Life Impact Scale (LLIS) and addressing fear of cancer recurrence as a potential psychosocial barrier to adherence [33]. Similarly, Sherman et al.’s therapeutic writing intervention (categorized as Mind–Body) targeted body image distress and body appreciation among a subgroup of breast cancer survivors with lymphedema [36]. These examples illustrate how, despite being theoretically driven, the limited overlap in outcomes constrains comparisons and highlights the need for more standardized theory-driven outcome selection to strengthen the evidence base for psychosocial interventions in lymphedema.

Methodologically, the studies in this sample demonstrated strong rigor in study design compared to those included in previous reviews [6, 11], although prior reviews not limited exclusively to intervention studies [44]. In contrast to earlier reviews reporting that most studies were pilot designs, only three studies in the current sample were described as pilot studies. The pilot studies in this review were conducted as preliminary investigations to evaluate the feasibility, safety, and acceptability of interventions, providing a foundation for future large-scale clinical trials [41]. While this suggests improved study maturity in the field, pilot studies often involve smaller sample sizes and limited statistical power [44]. Consistent with prior reports, seven of ten studies in our sample lacked adequate statistical power, often due to recruitment challenges or participant dropout. Limited power in our sample reflects broader trends in rehabilitation research, including the absence of power analysis, smaller-than-expected sample sizes, and funding constraints [44]. Finally, only 20% of interventions were conducted across multiple sites, highlighting a persistent limitation in lymphedema and rehabilitation research, a lack of large multi-site studies to generate robust evidence-based treatment protocols [43].

This systematic review combined methodological rigor and study effectiveness to examine the strength of the evidence for psychosocial interventions for individuals with lymphedema, making comparisons across intervention models and delivery settings. Comparisons by intervention model type were inconclusive due to the variety of intervention types and outcomes within each category. However, analyses comparing delivery settings suggest that interventions delivered in dual settings (in-clinic and remote) may be more effective than those delivered in a single setting. This finding should be interpreted with caution, given the very small number of studies in the review, and that the observed differences between the two settings may reflect confounding factors such as differences in population and intervention type. Nonetheless, this pattern is consistent with research on other types of interventions, such as therapy for mental health problems. For example, interventions that combine face-to-face and internet-based sessions of mental health treatment have demonstrated enhanced feasibility, reduced clinician time, lower dropout rates, and sustained therapeutic gains over time [45]. One such intervention (i.e., mobile phone intervention) in our review attributed improved adherence to the ability to address questions and anxieties in a timely manner [32]. Research on remote delivery, accelerated by the COVID-19 pandemic, suggests that patient preferences for delivery modalities vary based on demographic characteristics and clinical severity [46]. For example, the authors of a remote-only study in our sample reported that delivery setting preferences could vary by topic, with patients preferring limited face-to-face discussion for sensitive topics [36]. These findings suggest that hybrid delivery systems may capture the benefits of both delivery settings in improving psychosocial outcomes [45, 47].

## Limitations of the present review

Several limitations of this study should be considered. First, the search was limited to select databases and hand searching, and non-peer-reviewed gray literature reports were not included. Publications were also limited to those available in English, potentially excluding relevant international studies. Methodological quality ratings using the MQRS were subjective, yet biases were minimized by including two independent raters and a third rater to resolve disputes if necessary. Although this study used a well-established instrument to measure methodological rigor, using a mean split to determine high versus low rigor for each study may have underestimated the extent of variation between studies and may have led to less precise findings. Finally, because the instruments used to assess outcomes varied across studies and there were a limited number of studies, outcomes were categorized broadly as either mental health or quality of life measures. This may have resulted in conclusions that did not fully reflect the specific areas of impact of the interventions in this review.

## Conclusions & future research

Despite limited studies in this area, our review findings support the potential of psychosocial interventions to help improve mental health and quality of life among individuals with lymphedema. Intervention delivery settings appear to be important; the current review suggests that a combination of in-clinic and remote (IC&R) delivery settings may offer stronger support for improving outcomes than single-setting interventions. More research on psychosocial interventions using hybrid delivery settings is warranted to confirm their effectiveness. Second, consistency in outcome measures across studies (i.e., establishing a gold standard) for both quality of life and mental health would be useful for comparing intervention effectiveness. Although three of ten studies were pilot studies with small sample sizes and of lower methodological rigor, they reported significant improvements in outcomes. This suggests that these interventions are promising and merit future study with larger samples. Taken together, these findings support the utility of greater attention to developing and testing psychosocial interventions tailored for individuals with lymphedema throughout the lymphatic care continuum, with the ultimate goal of integrating these interventions into standard treatment to support the multidimensional needs of this population.

## Supplementary Information

Below is the link to the electronic supplementary material.

Supplementary file1 (DOCX 26 KB)

## Data Availability

Data are available upon request. Additional information available at Open Science Framework ID: osf.io/2szyv.

## Declarations

### Prior Presentation

This work was presented at the National Lymphedema Network (NLN) Annual Conference in Baltimore MD. July 2025.
