# Supplementary material for: Interventions to improve the psychosocial outcomes of individuals with lymphedema: a systematic review
Source: Support Care Cancer. 2026 Jul 3;34(7):724. doi: 10.1007/s00520-026-10972-9 (PMC13328148; doi:10.1007/s00520-026-10972-9)
Supplement: Supplementary file 1 — Supplementary file1 (DOCX 26 KB) [file 520_2026_10972_MOESM1_ESM.docx]

**Interventions to Improve the Psychosocial Outcomes of Individuals with Lymphedema:**

**A Systematic Review**

**Supplemental Tables/Figures**

*Table S1-* *Methodological Quality Rating Scale (MQRS) Item Rating by Study*

| **Authors (Year)** | **Intervention Type** | **Total** | **Study Design** | **Theoretical Foundation** | **Treatment Fidelity** | **Measures** | **Follow-Up** | **Dosage** | **Dropouts/** | **Blinding** | **Analyses** | **Power** | **Multi-Site** | **Generalizability** |
| --- | --- | --- | --- | --- | --- | --- | --- | --- | --- | --- | --- | --- | --- | --- |
|  |  |  |  |  |  |  |  |  | **Attrition** |  |  |  |  |  |
| **Abassi et al. (2018)** | **Mind-body** | **11** | **1** | **1** | **1** | **1** | **2** | **1** | **1** | **1** | **1** | **0** | **0** | **1** |
| **Sherman et al. (2018)** | **Mind-body** | **13** | **2** | **1** | **1** | **1** | **2** | **0** | **1** | **1** | **1** | **1** | **1** | **1** |
| **Loibnegger-Traußnig et al. (2023)** | **Mind-body** | **9** | **2** | **1** | **1** | **1** | **2** | **0** | **1** | **0** | **1** | **0** | **0** | **0** |
| **Omidi et al. (2020)** | **Education & Support** | **12** | **2** | **1** | **1** | **1** | **2** | **0** | **1** | **1** | **1** | **1** | **0** | **1** |
| **Okutsu & Koiyabashi (2014)** | **Education & Support** | **5** | **2** | **1** | **0** | **1** | **0** | **0** | **1** | **0** | **0** | **0** | **0** | **0** |
| **Ridner et al. (2020)** | **Education & Support** | **12** | **2** | **1** | **1** | **1** | **2** | **1** | **1** | **1** | **0** | **1** | **0** | **1** |
| **Arinaga et al. (2019)** | **Physical Activity** | **8** | **2** | **1** | **1** | **1** | **0** | **1** | **1** | **0** | **1** | **0** | **0** | **0** |
| **McClure et al. (2010)** | **Physical Activity** | **11** | **2** | **1** | **1** | **1** | **2** | **1** | **1** | **1** | **1** | **0** | **0** | **0** |
| **Loudon et al. (2014)** | **Physical Activity** | **12** | **2** | **1** | **1** | **1** | **1** | **1** | **1** | **1** | **1** | **0** | **1** | **1** |
| **Pasyar et al. (2019)** | **Physical Activity** | **8** | **2** | **1** | **1** | **1** | **0** | **1** | **1** | **1** | **0** | **0** | **0** | **0** |
| **Item Total** |  |  | **19** | **10** | **9** | **10** | **13** | **6** | **10** | **7** | **7** | **3** | **2** | **5** |
|  |  |  |  |  |  |  |  |  |  |  |  |  |  |  |

*Figure S2- MQRS rating chart for all included studies (N=10)*
